# Information Wanted

**Published:** 1884-07

**Authors:** 


INFORMATION WANTED.—We have before us a postal card containing a call for a meeting of the faculties of the Dental Colleges of the United States, to be held in New York City on August 4th, and also the notification that a preliminary meeting for conference will be held in the same city on *Sunday* afternoon, August 3rd, the same card being signed by the Dean of one of the Colleges as *Chairman*.

What we wish to know is by what authority a Chairman has been appointed prior to such a meeting being held; and also if there has already been a meeting of but a few of those interested, at which a course of action has been concocted?

We remember an "Association of Dental Colleges" in time past, which held several annual meetings, but which came to an untimely end by the refusal of a certain school in Philadelphia to depart from a rule it had established of graduating appliants on 12 years of practice and an examination.

If we were assured of the sincerity of this movement, and were convinced that any proper legislation by such a meeting would be strictly adhered to, nothing would give us greater pleasure than to participate. In our judgement any action determined upon at this meeting should be afterwards submitted to the faculties of the different dental institutions for their approval or otherwise.

The idea of one member of a faculty deciding for all of his colleagues, is indicative of something that had better be closely examined into before it is adopted.

